# Measuring the robustness of network community structure using assortativity

**DOI:** 10.1016/j.anbehav.2015.12.007

**Published:** 2016-02

**Authors:** Daizaburo Shizuka, Damien R. Farine

**Affiliations:** a School of Biological Sciences, University of Nebraska-Lincoln, NE, U.S.A.; b Edward Grey Institute of Field Ornithology, Department of Zoology, University of Oxford, U.K.; c Department of Anthropology, University of California Davis, CA, U.S.A.; d Smithsonian Tropical Research Institute, Panama

**Keywords:** bootstrapping, community detection, flock, golden-crowned sparrow, modularity, social network, thornbill, tit

## Abstract

The existence of discrete social clusters, or ‘communities’, is a common feature of social networks in human and nonhuman animals. The level of such community structure in networks is typically measured using an index of modularity, *Q*. While modularity quantifies the degree to which individuals associate within versus between social communities and provides a useful measure of structure in the social network, it assumes that the network has been well sampled. However, animal social network data is typically subject to sampling errors. In particular, the associations among individuals are often not sampled equally, and animal social network studies are often based on a relatively small set of observations. Here, we extend an existing framework for bootstrapping network metrics to provide a method for assessing the robustness of community assignment in social networks using a metric we call community assortativity (*r*_com_). We use simulations to demonstrate that modularity can reliably detect the transition from random to structured associations in networks that differ in size and number of communities, while community assortativity accurately measures the level of confidence based on the detectability of associations. We then demonstrate the use of these metrics using three publicly available data sets of avian social networks. We suggest that by explicitly addressing the known limitations in sampling animal social network, this approach will facilitate more rigorous analyses of population-level structural patterns across social systems.

## Highlights

• Measuring community structure in networks requires assigning nodes to communities.
• We propose a method to assess observation error in community assignments.
• We use simulations and empirical data to validate our robustness index (*r*_com_).
• We detail the interplay between sample size, modularity and community assignments.
• Our metric, *r*_com_, could accompany modularity values in studies of network structure.

Social network analysis has emerged as a useful method for quantitative analyses of complex systems including the structure of animal societies (Croft et al., 2008, Farine and Whitehead, 2015, Krause et al., 2007, Sih et al., 2009, Wey et al., 2008, Whitehead, 2008a). In particular, network analysis has been useful for understanding fission–fusion dynamics in which social aggregations of individuals (e.g. flocks, schools and herds) represent nonrandom subsets of larger social groups, or ‘communities’. In social networks that represent patterns of associations between individuals, social cohesion among subsets of individuals emerge as clusters of nodes that are tightly linked together (Kerth et al., 2011, Silk et al., 2014, Sundaresan et al., 2007). Variations in the patterns of clustering in social networks can arise from variations in the degree to which individuals show fidelity to a specific social community. At one extreme, associations may occur exclusively within social communities, producing a network consisting of a collection of independent social groups. At the other extreme, individuals may associate randomly (in which case assignments to communities would be arbitrary and meaningless), resulting in a network with little clustering. Many societies show intermediate patterns with relatively stronger associations within versus across social communities, for example when spatially discrete social groups are connected by individuals that affiliate with multiple groups. The pattern of community structure that emerges from nonrandom associations has widespread implications for evolution of cooperation (Marcoux and Lusseau, 2013, van Doorn and Taborsky, 2012), social selection (Farine and Sheldon, 2015, Formica et al., 2011), social communication (Bradbury & Vehrencamp, 2011), flow of information/disease (Adelman et al., 2015, Aplin et al., 2012, Onnela et al., 2011, Salathe and Jones, 2010) and the establishment and maintenance culture (Aplin et al., 2015).

While quantitative analyses of network structure present a powerful method to understand the socioecology of animals, the inferences we make about social dynamics often hinge on social network measures for which we cannot estimate robustness or uncertainty. Animal behaviourists have long been aware of the dangers of biased sampling design and the need to account for the possibility of errors in sampling that affect statistical results (Altmann, 1974). In social network analysis, the most common form of sampling error is estimate error arising from insufficient data collected when defining the relationships among all possible pairs of individuals (Farine and Strandburg-Peshkin, 2015, Farine and Whitehead, 2015). Incomplete sampling can easily affect the characterization of the global social structure of the study population (Kossinets, 2006, Lusseau et al., 2008). Incorrect networks can also arise if associations are defined without a clear understanding of the underlying social dynamics, as is the case when one infers social relations based on associations in groups (Farine, 2015, Farine and Whitehead, 2015, Franks et al., 2010, Whitehead and Dufault, 1999). Exhaustive sampling to generate weighted social networks will, in general, overcome issues of identification error and other types of sampling error (James, Croft, & Krause, 2009), whereas appropriate null models can account for any biases in the observation data (Farine & Whitehead, 2015). However, it is not always straightforward to assess the effects of sample size, and thus the potential impact of sampling error, on the precision of social network measures because these effects depend in part on the structure of the network itself (e.g. Whitehead, 2008b). Thus, robust methods that estimate uncertainty surrounding sampling effort when quantifying social network metrics greatly improve our inferences about social dynamics and structure of animal societies.

Resampling techniques such as bootstrapping (Efron & Tibshirani, 1994) have been proposed as approaches to evaluate uncertainty in social network analysis (Lusseau et al., 2008, Whitehead, 2008b) A bootstrapping procedure involves randomly resampling the data stream (i.e. the observation of groups across time) with replacement such that some groups (or distinct observations) are repeated multiple times, while others are not included. Relevant metrics can be calculated from this bootstrap replicate network, and the process can be repeated many times (e.g. 1000 times, each time sampling the data differently) to generate a confidence interval of the network metric for a given set of data. This resampling technique has been used effectively in various empirical studies to estimate uncertainty in network metrics assuming that the sample is unbiased (e.g. Gero et al., 2013, Shizuka et al., 2014).

In this study, we discuss some considerations that need to be taken into account when applying bootstrapping methods to assess the robustness of community structure in networks. We focus particularly on the robustness of ‘community assignment’, a key step in the process of estimating community structure whereby nodes on a network are partitioned into discrete communities based on their patterns of connectivity. Our confidence in community assignment depends on both the degree to which individuals associate within versus across communities (‘community fidelity’) and the degree to which our sampling is incomplete (‘sampling error’). Metrics of community structure such as modularity (see below) capture the degree of community fidelity when sampling is robust. Our goal is to develop a method to assess the influence of sampling error on community assignments, and provide a measure of certainty to accompany the modularity score *Q*. Our method combines bootstrapping with a coefficient of assortative mixing (Farine, 2014, Newman, 2002, Newman, 2003) to generate a single metric, which we call ‘community assortativity’ (*r*_com_). We then test our methods using simulations and provide several applications of our procedure to empirical datasets of avian social networks.

## Background

### Detection of Community Structure from Observation Data

Girvan and Newman (2002) first proposed a method for ‘community detection’, enabling the detection of unknown numbers of clusters within networks. This work initiated an explosion of studies on methods of partitioning networks into clusters of tightly linked nodes (i.e. sets of nodes that are more strongly connected to each other than they are to other nodes). There are now numerous methods for partitioning clusters on networks (Fortunato, 2010), and some of the most commonly used methods rely on the concept of modularity optimization. Modularity optimization techniques seek to partition a network in a way that maximizes the within-community rates of association or interactions. This maximum modularity value (*Q*) is the proportion of edges (or edge weights) that occur within communities relative to expected proportion of within-community edges if edges were distributed at random. This value is taken to be the measure of how much more community structure is present in the network compared to a random network with the same degree distribution. Importantly, the modularity value *Q* depends on the particular assignments of nodes into communities, and the robustness of the *Q* value also relies on the robustness of the assignments of nodes to communities.

### Bootstrapping to Measure Robustness of Community Structure

**Figure 1:**
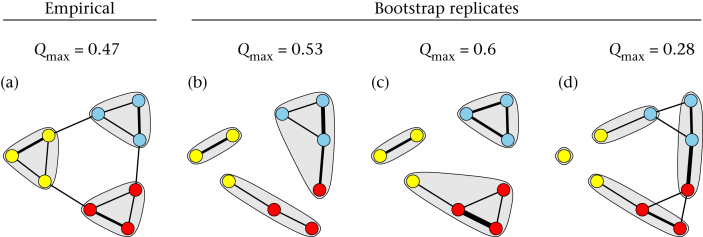
Applying community detection to bootstrap replicates can lead to misleading patterns. (a) A hypothetical empirical network with three communities, and (b–d) three sample bootstrap replicates of this hypothetical network. In each figure, the original community assignments are represented in node colours and the bubbles represent the communities identified by Clauset et al. (2004) algorithm in each network. Modularity values based on the communities represented by the bubbles are noted above each network. Note that bootstrap replicates (b) and (c) show a high degree of modularity, but the communities do not match with the empirical network. In bootstrap replicate (d), there are four communities (one isolate node forms its own community). Thus, bootstrapping leads to modularity values that are based on vastly different partitioning patterns.

Having measured community structure in a network using *Q*, the next step is to test whether this result is robust given the sampling effort. Lusseau et al. (2008) proposed that bootstrapping could be used to account for sampling error in estimating community structure: one could simply measure *Q* for each bootstrap replicate network and generate a confidence interval for the estimate of modularity. However, the confidence interval for the *Q* value generated by this bootstrapping procedure reflects the overall level of community structure per se, but does not represent confidence in the specific pattern of community structure (i.e. the assignments of individuals to different social communities). This is because applying the community detection anew to each bootstrap replicate often leads to different patterns of partitioning of the network (i.e. different numbers of clusters or the same number of clusters composed of different sets of nodes; Fig. 1). Yet, the particular membership of individuals in different social clusters is often the focus of social network research.

### Measuring Confidence in Community Assignments Using Assortativity

We propose that the bootstrapping approach can be extended to evaluate the confidence of the original partitioning of the network into communities. We can estimate the effect of sampling effort as the probability that a pair of nodes that are assigned to the same community in the empirical network will also be assigned to the same community in bootstrapped replicate networks. At the level of the whole network, we can assess the robustness of community assignments using an index called ‘assortativity’, which is a correlation coefficient that measures the association patterns between different types of nodes (Farine, 2014, Newman, 2002, Newman, 2003). We can use this coefficient of assortativity to measure the degree to which pairs assigned to the same community in the empirical network also occur in the same community in bootstrap replicate networks (see also Shizuka et al., 2014). Alternative indices for comparing community assignments (e.g. normalized mutual information: Danon, Diaz-Guilera, Duch, & Arenas, 2005) could also be used in a similar way to compare empirical and bootstrap replicate networks. Here, we describe the general method using the coefficient of assortativity, validate this method using simulations and provide empirical examples of its application to animal social networks.

## Methods

### Network Construction and Community Detection

Our method is applicable to networks captured using the ‘gambit of the group’ method in which groups (flocks, herds, etc.) are observed across time to produce a data stream (this is the case for the empirical networks described below) or networks based on observations of interactions or associations between pairs of individuals (dyads). The data stream of observations can then be used to calculate pairwise association indices (e.g. using the simple ratio index; Cairns & Schwager, 1987). In the resulting social network, nodes represent individuals and edge weights represent pairwise association indices.

In all networks (simulated and empirical; Fig. 1), we used the Clauset, Newman, and Moore (2004) community detection algorithm using the ‘fastgreedy.community’ function in igraph v.0.6 (Csardi & Nepusz, 2006), although the general method could be applied to any community detection method. Using this method, we assigned each node to a cluster or community and measured the modularity value *Q* of the proportion of edge weights that occurred within communities relative to random expectation. For visualizations of the network, the resulting community assignments in the observed networks (again, for both simulated and empirical) in are represented by different colours of nodes (see Results, Fig. 4).

### Calculating *r*_com_ from Bootstrap Replicates

In the bootstrapping procedure, we resampled observations of groups (although one could also resample individual observations) with replacement to generate a replicate data stream of the same sample size. We built a ‘bootstrap replicate network’ using pairwise association indices and applied the same community detection algorithm (Clauset et al., 2004) to this network to assign individuals to communities. We then constructed an *n* × *n* matrix, **M**, where the cell value *M*_*ij*_ was 1 when nodes *i* and *j* were assigned to the same community and 0 otherwise. We simultaneously constructed a ‘co-presence’ matrix, **C**, in which *C*_*ij*_ = 1 when both nodes *i* and *j* existed in the bootstrap replicate, and 0 if *i* or *j* was missing from the replicate sample.

We then constructed a new *n* × *n* matrix of community co-membership, ***P***, that summarized the proportion of all bootstrap replicates in which the nodes *i* and *j* were both sampled and assigned to the same community. We defined the cell values of ***P*** as:

(1) $$P_{ij}=\sum \frac{M_{ij}}{C_{ij}}$$

Thus, the cell values *P*_*ij*_ represented the proportion of bootstrap replicates in which nodes *i* and *j* were assigned to the same community, given that both nodes were included in the replicate network.

Next, we measured the assortativity of *P*_*ij*_ values based on the original community assignments of nodes. Following notation similar to Newman (2003) and Farine (2014), the coefficient of assortativity by community assignment is:

(2) $$r_{\text{com}}=\frac{\sum_{x}e_{xx}-\sum_{x}a_{x}b_{x}}{1-\sum_{x}a_{x}b_{x}}$$

where *e*_*xx*_ is the proportion of the network edge weights that connect nodes that are assigned to community *x* in the empirical network, $a_{x}=\sum_{x}e_{xy}$ is the proportion of the network edge weights that start at nodes in community *x* and end at a node in community *y*, for all values of *y*, and $b_{x}=\sum_{y}e_{yx}$ is the proportion of network edge weights that begin at a node in community *y* and end at a node in community *x*, for all values of *y*. While the above equation is formulated for directed networks, it can be applied to undirected networks as well (Newman, 2003). When applied to the *P*_*ij*_ matrix, *r*_com_ represents the degree to which the proportion of community co-membership in bootstrap replicates occurs between nodes assigned to the same empirical community, compared to the random expectation (in this case nodes are applied to communities at random). The value of *r*_com_ is 1 when all bootstrap replicates result in the exact same community assignments as the empirical network. Conversely, *r*_com_ approaches 0 when community assignments in bootstrap replicates are random with respect to the original empirical communities (but we show below that values in random networks are generally greater than 0). The value of *r*_com_ can be negative if nodes assigned to different empirical communities are more often assigned to the same community in bootstrap replicates.

### Simulating Networks

We tested how *r*_com_ quantifies patterns of clustering under different levels of social structure by using simulated networks. In our simulations, each of *n* nodes was randomly assigned membership to one of *c* communities with equal probability. We generated data streams that consisted of sampling periods (Whitehead, 2008a), which are replicated binary networks that define whether a dyad was observed together or not during that sample. These were generated as follows.

(1) We set the population-level of community fidelity (the proportion of the edge weights among individuals within community, defined as *p*_*w*_).
(2) Because individuals often vary in their degree of attachment to communities, we multiplied the population-level community fidelity with a random value drawn from a normal distribution (mean = 1, standard deviation = 0.1) to create individual-level community attachment values, *p*_*wi*_. Resulting values that exceeded 1 were set to 1. We also created a measure of the proportion of individuals' associations outside their community, defined as *p*_*bi*_ = 1 − *p*_*wi*_.
(3) We created a ‘true’ underlying network by calculating the edge weight for each pair of individuals. Edges between individuals in the same community (*c*_*i*_ = *c*_*j*_) were defined as the product of the individual-level community attachment values (*e*_*ij*_ = *p*_*wi*_ × *p*_*wj*_). Similarly, edges connecting individuals between communities were weighted using the product of their propensity to associate outside of their community (*e*_*ij*_ = *p*_*bi*_ × *p*_*bj*_). Thus, these edge weights represented the underlying probabilities that two individuals in a population associated with each other.
(4) We introduced a global measure of how well the network was sampled (*p*_obs_ < 1). If *p*_obs_ = 1, then all edges were sampled in a given sampling period. If *p*_obs_ = 0.05, then only 1 in 20 edges were sampled in each sampling period. Thus, the probability that a given pair of nodes was observed associating in a given sampling period was *p*_obs_ × *e*_*ij*_.
(5) We simulated 100 sampling periods. We first created a three-dimensional matrix (*n* × *n* × 100) of associations forming our ‘observed’ data. In each sampling period, the *n* × *n* matrix represented observed associations during a time period (1, if a pair associated, and 0, if they did not). We then aggregated the associations across the 100 simulated sampling periods and constructed the observed social networks using the ‘get_network’ function in the R package asnipe (Farine, 2013). Edge weights were calculated using the simple ratio index.

We performed community detection on each of these simulated ‘observed’ social networks, and performed our bootstrapping method (resampling the sampling periods; Whitehead, 2008b) to calculate *r*_com_ as described above. For the simulated networks, we used 100 bootstrap replicates. We repeated this for 100 replicated networks. In each set of replicated networks, we varied population size (*n* = 10, 20, 30 and 40 individuals), the numbers of communities (*c* = 2, 3, 4 and 5 communities), population-level community fidelity (*p*_*w*_ = 0 to 1, at intervals of 0.05), and the level of sampling effort (*p*_obs_ = 0.05, 0.2, 0.4 and 0.8).

### Empirical Examples

We used three data sets as examples of the application of our robustness metric. We chose these three examples because we were involved in the data collection (thus we have reliable insights into the data collection methods), and they ranged in level of community structure. Note that these three data sets vary in data collection methods, spatial and temporal scales and the number of species observed. Only one of these data sets (Shizuka et al., 2014) was designed to study network community structure of a single species, while the other two studies observed mixed-species flocks.

Farine, Garroway, and Sheldon (2012), herein the ‘tit data’, collected a data set consisting of 151 individuals of five passerine species in Wytham Woods, U.K.: 78 blue tits, *Cyanistes caeruleus*, 7 coal tits, *Periparus ater*, 51 great tits, *Parus major*, 11 marsh tits, *Poecile palustris*, 3 Eurasian nuthatches, *Sitta europaea*, and 1 individual of unknown species. Individuals were all fitted with individually encoded passive integrated transponder (PIT) tags (IB Technology, Aylesbury, U.K.) that were logged by radio frequency identification (RFID) antennae (Dorset ID, Aalten, The Netherlands) fitted to each hole on regular sunflower feeders (we used unhusked sunflower seed). Data were collected from four feeders spaced approximately 300 m apart over four consecutive weekends (2 days each for a total of 8 days in January 2012). Feeders logged the presence of individuals at a sub-second resolution, and detections were assigned to flocks using a machine learning algorithm (based on Gaussian mixture models; Psorakis, Roberts, Rezek, & Sheldon, 2012). This algorithm identifies peaks in activity (the number of detections) to extract detections that are more clustered together in time than they are to other detections (hence detecting ‘flocks’, and thus our network inference is based on the ‘gambit of the group’ approach). This approach has been shown to be more robust than alternative methods for extracting data (Psorakis et al., 2015). We used the first day of data that is contained within the freely available R package asnipe (Farine, 2013).

Shizuka et al. (2014), herein the ‘sparrow data’, studied the community structure of flocks in a wintering population of a migrant sparrow, the golden-crowned sparrow, *Zonotrichia atricapilla*. This sparrow data set (available on Dryad: http://dx.doi.org/10.5061/dryad.d3m85) includes observations of flock membership of individually marked birds at a study site (ca. 7 ha) in Santa Cruz, California, U.S.A. during October–March in three seasons in 2009–2012. The social network showed high degrees of community structure in which three social communities with relatively discrete spatial home ranges occurred in all three seasons. The authors showed that individuals returning to the population across years were highly faithful to the social community. The measurement of robustness of community structure estimate using the *r*_com_ index for this data set is included in the Supplemental Information of the original study. Here, we present the results for one of the study seasons (October 2011–March 2012).

Farine and Milburn (2013), herein the ‘thornbill data’, collected repeated observations of 63 colour-marked passerines forming mixed-species flocks over a 67 ha area at Mulligan's Flat Nature Reserve, outside of Canberra, Australia. The data consist of 2 scarlet robins, *Petroica boodang*, 13 striated thornbills, *Acanthiza lineata*, 26 buff-rumped thornbills, *Acanthiza reguloides*, 14 yellow-rumped thornbills, *Acanthiza chrysorrhoa*, 4 speckled warblers, *Chthonicola sagittatus*, 2 white-throated treecreepers, *Cormobates leucophaea*, and 1 white-eared honeyeater, *Lichenostomus leucotis*. Daily observations of individual membership in flocks were recorded over 2 months (May and June 2011) using the gambit of the group (individuals observed together in a flock were assigned to the same unique flock number). The raw observation data for this study is provided as Supplementary Material.

For each empirical data set, we calculated *r*_com_, modularity (*Q*) and the confidence intervals of *Q* using Lusseau et al.'s (2008) method. In addition, we used a popular permutation test (Whitehead, 2008a) for comparing network metrics to random networks while controlling for the sampling method. This test enabled us to deal with known and unknown biases in the data, such as whether degree was correlated with the number of observations in the data to evaluate whether the value of *Q* itself was a meaningful test. Briefly, the permutation swaps observations of pairs of individuals between groups to increasingly randomize the observed data (Bejder, Fletcher, & Brager, 1998). After each swap, the associations between all individuals (i.e. edge values) can be remeasured and the network metric of interest (in our case *Q*) can be recalculated. If the patterns in the observed network differ significantly from random, that is, the population is more structured than expected given the observation data, then the value of *Q* from the observed network should fall outside of the 95% range of *Q* values (i.e. *Q*_rand_) calculated from the networks based on permuted data. We calculated *P* value of the *Q* statistic using permutations as described by Whitehead (2008a) and implemented in the asnipe package. We used the same method to construct and visualize all three networks: edge weight represented the simple ratio association index; there was no edge filtering; and networks were plotted using a force-directed algorithm with weighted edges (Fruchterman & Reingold, 1991).

### Simulations to Test Relationships Between *Q*, *r*_com_ and Sample Size

Because our goal was to provide a metric of robustness of measures of community structure, we investigated how *Q* and *r*_com_ values would have changed with sample size in our empirical networks. We predicted that the value of *r*_com_ would generally increase with sample size when the network exhibited structure, but that this relationship between sample size and *r*_com_ should depend on the underlying structure. When communities are very discrete (*Q* value is large), we expected *r*_com_ to be high even at modest sample size because associations across communities are rare. When communities are discrete but associations across communities are still common (*Q* value is intermediate), increasing sample size should lead to concordant increase in *r*_com_ because sampling effort could affect the relative frequencies with which we observe associations across communities. When *Q* value is small, increasing sample size may not increase *r*_com_ because there is little community structure (i.e. there is no pattern to detect).

We tested these ideas using our empirical networks that showed high, intermediate and low values of *Q* (tit network, sparrow network and thornbill network, respectively). For each network, we randomly subsampled a varying sample size of flock observations and calculated *Q* and *r*_com_ as explained above. We used random subsets of increasing sample size starting with *n* = 50 observations and increasing by 10 observations (*n* = 60, 70, etc.) up to the total number of observations for each data set. We conducted 50 replicate simulations at each sample size.

## Results

### Simulation Results

**Figure 2:**
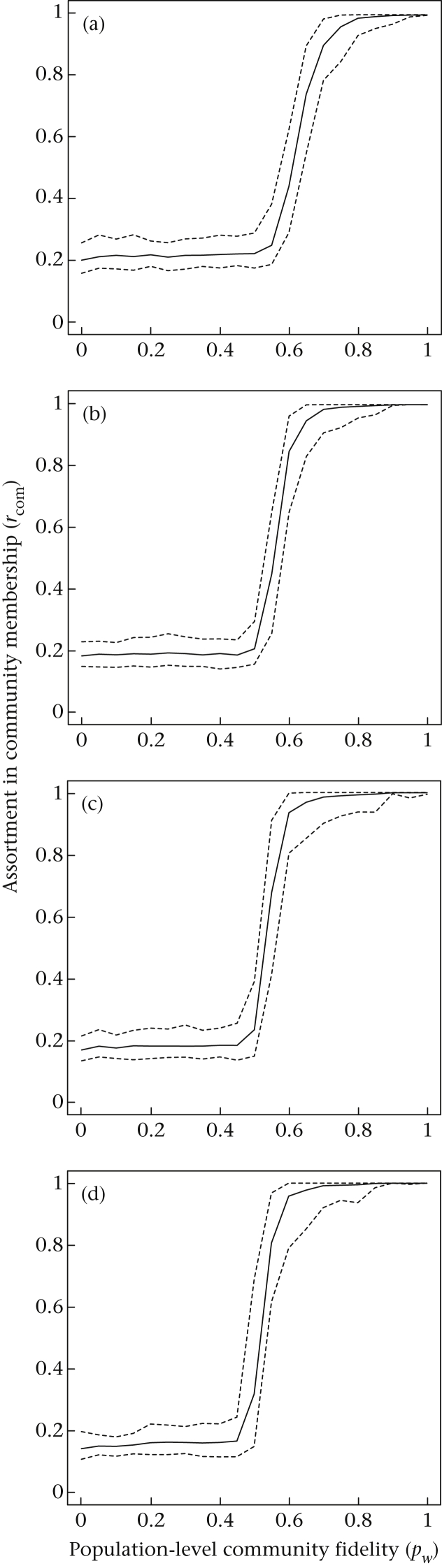
Assortment in community structure increases sharply at high levels of fidelity to communities (*p*_*w*_) and this is robust to probability of observation. Solid lines represent mean values, and slashed lines delineate 95% confidence intervals of *r*_com_ values in simulated networks with *n* = 40 nodes, *c* = 4 communities and (a) *p*_obs_ = 0.05, (b) *p*_obs_ = 0.2, (c) *p*_obs_ = 0.4 and (d) *p*_obs_ = 0.8.

**Figure 3:**
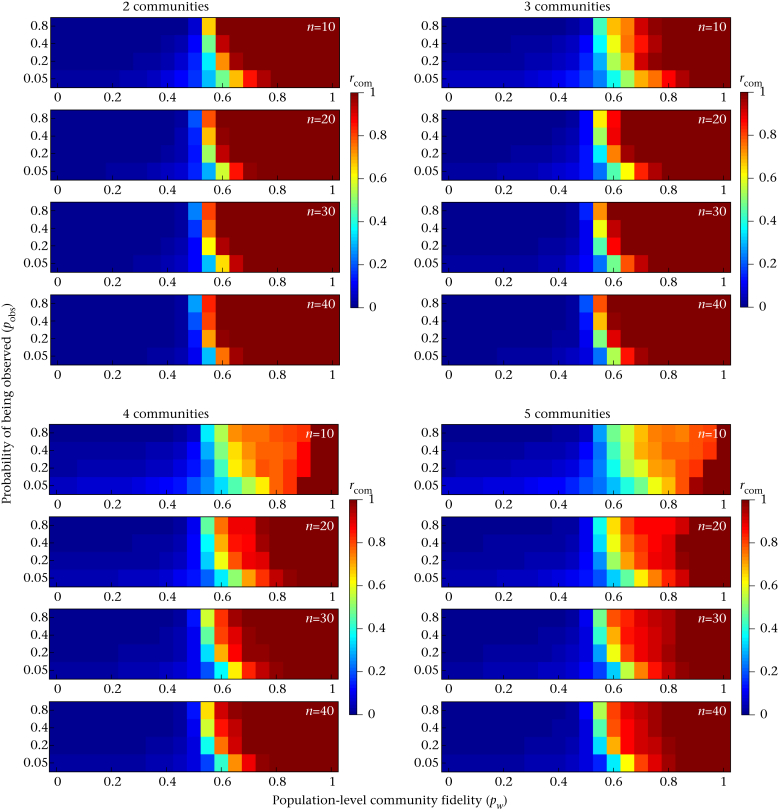
Full simulation results showing how *r*_com_ values are informative across different population sizes (*n*), numbers of communities (*c*), probability of being observed (*p*_obs_) and the fidelity to the community (*p*_*w*_).

As predicted, there was a strong relationship between the probability that individuals would associate with other members of their own community and the assortativity of nodes across the bootstrap replicates (Fig. 2). This relationship was nonlinear, with the robustness of community assignments rising rapidly as the probability that an individual associated with other members of their own community increased (*p*_*w*_ >> 0.5). In our simulations, community assignments seemed to be very robust (*r*_com_ > 0.9) with moderate levels of fidelity to communities (0.6 < *p*_*w*_ < 0.8, note here that decreasing values of *p*_*w*_ in our simulations were associated with an increasing probability of associating outside of the community *p*_*b*_, rather than simply a decrease in the probability of being observed). Moreover, *r*_com_ behaved consistently for networks of different sizes and different numbers of communities (Fig. 3), making this a very useful metric. Across all network sizes and numbers of communities that we investigated, there was a clear relationship between the probability of observation and robustness of community assignment: the robustness of community assignment was systematically lower when individuals were more difficult to detect.

Our simulations showed that *r*_com_ generally did not decline to zero but rather remained at low values (*r*_com_ ∼ 0.2) even when associations occurred randomly or even disassortatively with respect to original community assignments (*p*_*wi*_ ≤ 0.5; Figure 2, Figure 3). This is because the community detection procedure groups individuals together that are ‘observed’ relatively more often, even when associations occur randomly. This means that we often detected weak community structure independent of the ‘true’ (i.e. preassigned) community identities in these simulations. Then, given that bootstrapping only permutes the observations that exist, nodes that are observed together more often in the original network would be slightly more likely to be observed together in bootstrap replicates, generating a slightly positive *r*_com_ value. This actually simulates a real-life situation: we do not generally know what the ‘true’ community identity of any given individual is, and we can only infer community structure based on the observations that we make. Furthermore, individuals often vary in how many times they were observed, potentially generating similar patterns. This highlights the need to take some care when evaluating *r*_com_ as small values may not represent ‘near significance’, and we recommend using the permutation test to evaluate the validity of the *Q* parameter against biases in the data. In general, only large values (roughly >0.5, based on our simulations) should be deemed as robust evidence for structure. However, given how rapidly this metric approaches 1, interpretation should rarely be a problem in well-sampled networks.

### Empirical Results

We used three empirical data sets as examples of applying the bootstrap technique to measure community assortativity to animal social networks. The data sets represent three different levels of community structure, which probably arose from the different ecologies of the study systems.

**Figure 4:**
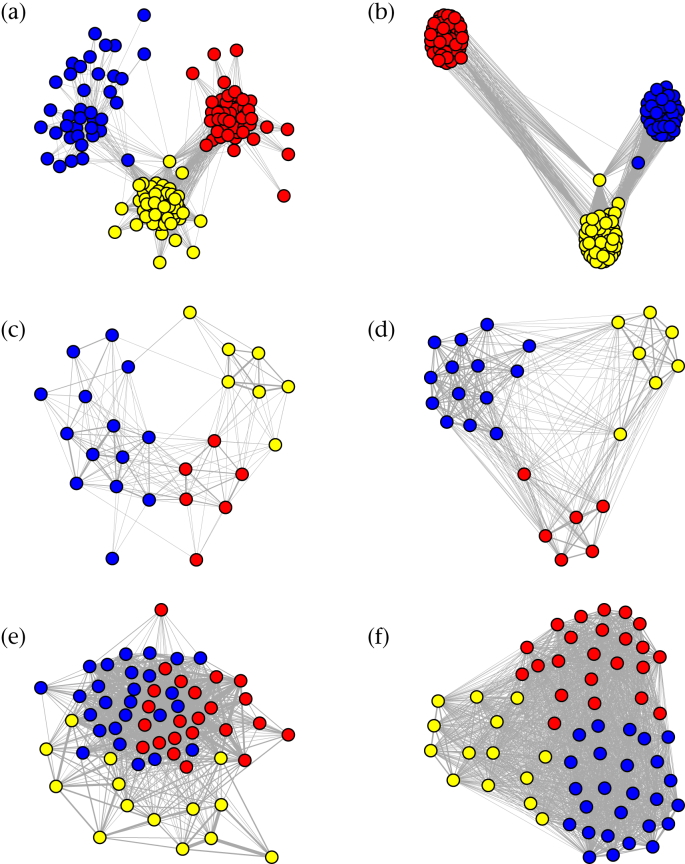
Three empirical examples showing a range of community structure. (a, c, e) Empirical social networks where edge widths represent the pairwise association index and node colours represent assignments into social communities using the Clauset et al. (2004) algorithm. (b, d, f) Networks in which nodes represent the same individuals and edges represent the number of times a pair of nodes was assigned to the same community in bootstrap replicate networks using the same community detection algorithm. (a, b) Mixed-species ‘tit network’ (Farine et al., 2012); (c, d) social network of golden-crowned sparrows (Shizuka et al., 2014); (e, f) mixed-species flock of thornbills (Farine & Milburn, 2013). Figures may appear different from those presented in the original studies because we imposed uniform criteria for layout (Fruchterman-Reingold method with edge weights), edge widths and edge filtering (no filter). (a, b) A single isolate node is removed for clarity. (c, d) Individuals observed fewer than three times were removed from the network, as in the original publication (Shizuka et al., 2014).

For each of the three networks, the Clauset et al. (2004) algorithm identified three social communities, and all networks had higher modularity than expected from randomized networks (Table 1). However, the level of community structure varied considerably (Fig. 4). The relative values of modularity and *r*_com_ (Table 1) tell the same story: the data set on mixed flocks of tits (Farine et al., 2012) showed the strongest level of community structure *Q*, and the most robust data based on *r*_com_. The social structure of wintering golden-crowned sparrows (Shizuka et al., 2014) also showed relatively strong community structure, with intermediate values of *Q*, but the lower value of *Q* likely arose primarily from the propensity of some individuals to associate with multiple communities (community fidelity) rather than from sampling effort, given that *r*_com_ was relatively large. The mixed-species flocks of thornbills (Farine & Milburn, 2013) showed the weakest level of community structure (low *Q*), and the communities that were detected were only marginally robust (*r*_com_ is near 0.5). These results seem to be in accordance with the findings in the original studies: Farine et al. (2012) measured associations in three discrete locations, and only 32% of individuals were detected at more than one location; Shizuka et al. (2014) observed distinct spatial boundaries between flock home-ranges, whereas Farine and Milburn (2013) reported very little spatial segregation among home ranges of individuals in their study.

**Figure 5:**
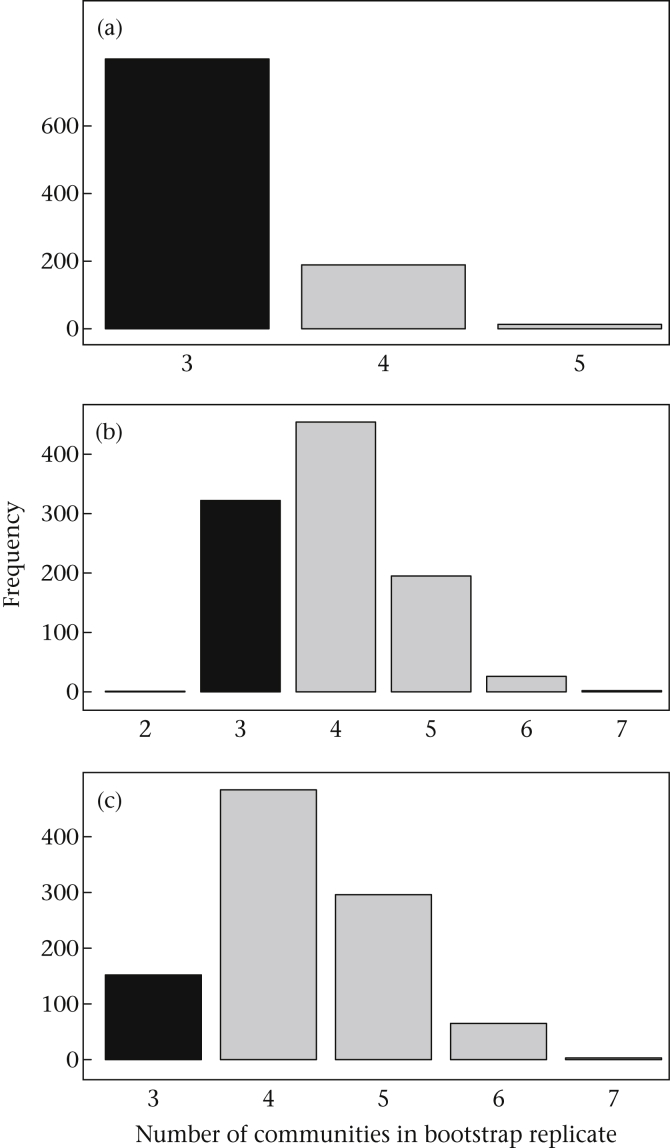
Number of communities identified in bootstrap replicate networks can differ from empirical network. Histogram of numbers of communities in bootstrap replicates, with the empirical number of communities shown in black (*c* = 3 in all examples), for the (a) tit network, (b) sparrow network and (c) thornbill network. (a) When empirical community assignments are very robust, as in the tit network, bootstrap replicates often match the empirical network. (b, c) However, when robustness of empirical community assignments is low, bootstrap replicates may be partitioned into different numbers of communities, even in the sparrow network (b).

While the confidence intervals of each network encompasses the empirical values and appear to be reasonable (Table 1), examining our results in more detail suggests that these confidence intervals could be misleading. Fig. 5 shows the number of communities identified in bootstrap replicate networks using the same Clauset et al. (2004) algorithm. In the tit mixed-species flock network, the majority of bootstrap replicates contained the same number of social communities as the empirical network, reflecting the high degree of robustness of the empirical community structure. However, in both the sparrow and thornbill networks, the majority of the bootstrap replicates contained different numbers of communities than the empirical network. That is, the estimates of modularity in bootstrap replicates were based on partitioning patterns that differed from the empirical network. A likely explanation is that bootstrap replicates tended to exclude weak edges based on very few observations and created isolated nodes or clusters, increasing the number of communities (see Fig. 1 for hypothetical examples). More importantly, both the confidence intervals generated from bootstrapping and the *P* values generated from the permutation test estimated that the thornbill network had significant community structure, contrasting with the estimate from *r*_com_ and the visual evidence when plotting the network (Table 1, Fig. 4). This is likely due to a general property of social networks: significant clustering patterns often arise when some individuals maintain overlapping home ranges even in the absence of social communities.

Our simulations using subsets of the data reveal how the values of *Q* and *r*_com_ differ with both the community structure of the network and the sample size. The inferred structure of the tit network was robust even at modest sample size (e.g. average *r*_com_ = 0.94 with *n* = 50 observations), and adding more observations did not change the estimate of *Q* nor the robustness of this estimate. In contrast, the *r*_com_ value increased with sample size for the sparrow network while the *Q* value remained relatively constant. This reflects the fact that increasing sample size increases our confidence that the inferred community structure is correct. Finally, in the thornbill network, the *r*_com_ value was uniformly low regardless of increasing sample size. This reflects the fact that, when there is weak community structure in the network, increasing the sample size does not make assignments of nodes to communities more robust. Also note that the average *Q* value decreased with sample size in the thornbill network, which suggests that the *Q* value itself may not be robust in this network.

## Discussion

We propose that *r*_com_, an index of assortativity of community assignments in bootstrap replicates, is a useful way to assess the robustness of empirical measurements of community structure. We confirmed through simulations (Figure 2, Figure 3) that this index is useful in systems of different sizes (range 10–40 nodes) and different numbers of communities (range 2–5 communities). The detectability of individuals has some bearing on the robustness of our estimates of community structure, probably because low detectability lowers the sample size of observed associations. This highlights the need to take care when inferring networks from sparse observation data and to evaluate the robustness of network metrics.

Our work extends the bootstrapping method proposed by Lusseau et al. (2008) to account for uncertainty in social network data. While confidence intervals for modularity values can be generated as advocated by Lusseau et al. (2008), we have shown that applying community detection methods to bootstrap replicate networks often results in partitions that do no match the community structure identified in the empirical network, (Figure 1, Figure 5). Thus, the confidence interval generated by bootstrapping is not an appropriate way to assess error in the modularity value given a particular pattern of partitioning. Our measure, *r*_com_, circumvents this problem by directly assessing the degree to which empirical community assignments of nodes agree with community assignments in bootstrap replicates.

**Figure 6:**
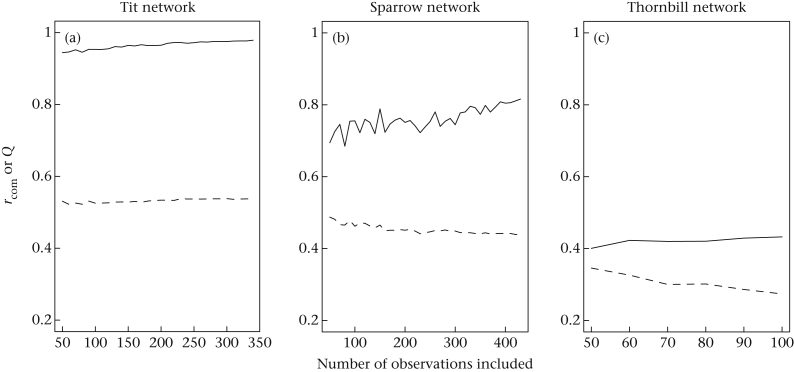
Simulations using subsamples of varying numbers of observations show that *r*_com_ increases with sample size but *Q* does not. In each panel, lines show the changes in the average *r*_com_ value (solid line) and average *Q* value (dashed line) with sample size. We randomly subsampled each data set using different sample sizes and calculated the means values from 50 replicate simulations.

Qualitatively, the reliability of community assignments correlates with modularity (Table 1), but the relationships between *Q*, *r*_com_ and sample size can be complex. At the extremes of clustering patterns, *r*_com_ will be relatively independent of sampling effort: community assignments will be robust when communities are very discrete (e.g. tit network; Fig. 6a), and community assignments will not be robust when there is little community structure (e.g. thornbill network; Fig. 6c). However, in systems with intermediate levels of community structure (medium *Q* values), *r*_com_ will be positively correlated with sampling effort (e.g. sparrow network; Fig. 6b), reflecting the fact that increased sampling improves the robustness of community assignments. Thus, although *r*_com_ is not itself a statistic of the network, it can facilitate interpretation of community structure. More generally, our study adds to the evidence that the robustness of network metrics depends not only on sample size but also on the underlying structure of the network (Whitehead, 2008b).

Our general approach to using assortativity may provide new avenues to address other questions about social dynamics in networks. For example, assortativity could be used to measure changes in community membership across time (sensu Mucha et al., 2010, Tantipathananandh and Berger-Wolf, 2011). While changes in modularity across time would reflect changes in overall social structure, it would not necessarily capture the dynamics of community membership (e.g. same community structure persists, but membership changes across time). However, our measure of community delineation could be extended to explicitly test for such changes in community structure within social networks. To accomplish this, equation (2) can be restated to the proportion of edges that are in the same community across two network samples. This would determine whether membership to communities has changed independently of any changes (or lack thereof) in the global network structure. We suggest that assortativity may be a generally useful metric for studies of community structure in social networks.

Our method provides a robust estimate of the uncertainty surrounding the assignment of individuals into distinct communities in the same way as correlation coefficients do: the assortativity coefficient *r* is based on the Pearson's correlation coefficient. In doing so, it goes some way to addressing the broader issues with estimating uncertainty in the observed network structure (Farine & Whitehead, 2015). We believe that this general approach can be applied to other network metrics. However, this approach still relies on a network based on observations of individuals, or pairs of individuals, that are unbiased (i.e. no systematic preference towards observing certain categories of individuals) and that contains only random sampling noise (which our simulations show have little impact on *r*_com_). Thus, it should not be substituted for continued research effort to find tools to estimate the uncertainty of observed network and network edges (Farine & Strandburg-Peshkin, 2015) and using permutation tests to evaluate the statistical significance of effect sizes (Farine & Whitehead, 2015).

In this study, we chose three avian data sets that represented a spectrum of community structure and for which we had access to the full data stream. These study systems and data collection methods have important differences that contribute to the observed social network structure, such as species composition (multiple versus single species), data sampling technique (PIT-tag feeders versus observation of marked individuals) and spatial scale. Thus, the approaches described herein can be applied to systems that differ in many ways, and our study clearly demonstrates the utility of incorporating estimates of robustness to accompany community delineation measures such as modularity. With the continued increase in the number of animal social network studies, this will offer many opportunities for testing the ecological and evolutionary underpinnings of animal societies. The rich literature on network analysis offers many more possible avenues for estimating uncertainty in network data (e.g. Clauset et al., 2008, Guimera and Sales-Pardo, 2009, Handcock et al., 2007), and some of these methods may also prove useful to animal behaviourists. Further development of reliability estimates for network metrics will be key to progressing beyond static networks to investigate temporal dynamics in network structure, and ultimately to facilitate comparative analyses within and between study systems.

## Figures and Tables

**Table 1:** Summary of results of empirical networks

Data set	*n*	No. of groups	*c*	*Q* (CI)	*P*	*r*_com_
Tits	151	347	3	0.54 (0.52–0.59)	<0.001	0.99
Sparrows	27	430	3	0.43 (0.37–0.50)	<0.001	0.81
Thornbills	63	109	3	0.22 (0.20–0.34)	0.001	0.46

Here, *c* is the reported number of communities from the detection algorithm, *Q* is the modularity index with 95% confidence intervals (CI) estimated using a bootstrap test, *P* is the significance estimated from a standard data permutation test (evaluating whether *Q* is larger than expected based on shuffling the observed data) and *r*_com_ is the assortativity index using the method described in this paper. Details of each method are given in the main text.
